# Prenatal maternal and cord blood vitamin D concentrations and negative affectivity in infancy

**DOI:** 10.1007/s00787-021-01894-4

**Published:** 2021-10-18

**Authors:** Sara Sammallahti, Elisa Holmlund-Suila, Runyu Zou, Saara Valkama, Jenni Rosendahl, Maria Enlund-Cerullo, Helena Hauta-alus, Marius Lahti-Pulkkinen, Hanan El Marroun, Henning Tiemeier, Outi Mäkitie, Sture Andersson, Katri Räikkönen, Kati Heinonen

**Affiliations:** 1 Department of Child and Adolescent Psychiatry/Psychology, Erasmus MC, Sophia Children’s Hospital, Rotterdam, The Netherlands; 2 Department of Social and Behavioral Science, Harvard T.H. Chan School of Public Health, Boston, MA USA; 3 Pediatric Research Center, Children’s Hospital, University of Helsinki and Helsinki University Hospital, Helsinki, Finland; 4 Research Program for Clinical and Molecular Metabolism, Faculty of Medicine, University of Helsinki, Helsinki, Finland; 5 Folkhälsan Research Center, Helsinki, Finland; 6 National Institute for Health and Welfare (THL), Helsinki, Finland; 7 PEDEGO Research Unit, MRC Oulu, Oulu University Hospital and University of Oulu, Oulu, Finland; 8 Department of Psychology and Logopedics, Faculty of Medicine, University of Helsinki, Helsinki, Finland; 9 Department of Psychology, Education and Child Studies, Erasmus School of Social and Behavioural Sciences, Erasmus University Rotterdam, Rotterdam, The Netherlands; 10 Psychology/Welfare Sciences, Faculty of Social Sciences, Tampere University, 33014 Tampere, Finland

**Keywords:** Vitamin D, Prenatal, Temperament, Negative affectivity, Infant mental health, Nutrition

## Abstract

Higher maternal vitamin D concentration during pregnancy is associated with better child mental health. Negative affectivity, an early-emerging temperamental trait, indicates an increased risk of psychopathology. We investigated if maternal early/mid-pregnancy 25-hydroxyvitamin D (25(OH)D) and neonatal cord blood 25(OH)D concentrations are associated with Negative affectivity in infancy. We studied term-born infants from the vitamin D Intervention in Infants study (VIDI, *n* = 777, follow-up rate 80%, Finland), and the Generation R Study (*n* = 1505, follow-up rate 40%, Netherlands). We measured maternal serum 25(OH)D at 6–27 weeks (VIDI) or 18–25 weeks (Generation R) of pregnancy, and cord blood 25(OH)D at birth (both cohorts). Caregivers rated infant Negative affectivity at 11.7 months (VIDI) or 6.5 months (Generation R) using the Revised Infant Behavior Questionnaire. Using linear regression, we tested associations between 25(OH)D and Negative affectivity adjusted for infant age, sex, season of 25(OH)D measurement, maternal age, education, smoking, and body-mass-index. Per 10 nmol/l increase in maternal early/mid-pregnancy 25(OH)D, infant Negative affectivity decreased by 0.02 standard deviations (95% confidence interval [CI] − 0.06, − 0.004) in VIDI, and 0.03 standard deviations (95% CI − 0.03, − 0.01) in Generation R. Cord blood 25(OH)D was associated with Negative affectivity in Generation R (− 0.03, 95% CI − 0.05, − 0.01), but not VIDI (0.00, 95% CI − 0.02, 0.02). Lower maternal 25(OH)D concentrations were consistently associated with higher infant Negative affectivity, while associations between cord blood 25(OH)D concentrations and Negative affectivity were less clear. Maternal vitamin D status during early- and mid-pregnancy may be linked with early-emerging differences in offspring behavior.

**Supplementary Information:**

The online version contains supplementary material available at 10.1007/s00787-021-01894-4.

## Introduction

Vitamin D is an essential micronutrient and neurosteroid that can influence fetal brain development [1–3]. During gestation, the fetus cannot synthesize vitamin D, but rather maternal vitamin D is transported across the placenta, which may also play an active role in the metabolism of this prohormone into its active form [1, 3–5]. Since fetal vitamin D status depends on maternal supply, maternal vitamin D concentrations can be seen as the determinant or proxy of the closely correlated fetal vitamin D status [1, 3, 4]. Further, neonatal vitamin D concentrations measured from cord blood at birth can be used as a marker of fetal vitamin D status at the end of gestation [1, 4].

Vitamin D insufficiency is common and can have long-lasting implications for the offspring. Worldwide, one in every two pregnant women are estimated to have vitamin D insufficiency (serum 25-hydroxyvitamin D (25(OH)D) < 50 nmol/L), however the cut-offs used to define deficient or optimal levels of 25(OH)D often vary between studies [1]. A recent meta-analysis found evidence for associations between maternal prenatal vitamin D status and child neurobehavioral outcomes to be mixed. [6] Some, but not all [7, 8] previous studies have reported that low maternal vitamin D concentrations during pregnancy are associated with an increased risk of mental health problems in the offspring, including behavioral problems and diagnosis of attention deficit/hyperactivity disorder (ADHD), autism spectrum disorder (ASD), and depression. [9–15] Lower cord blood 25(OH)D concentrations at birth have also been associated with poorer mental health outcomes [13, 16–18], however, these may be less predictive of child neurobehavioral outcomes, compared to early- and mid-pregnancy maternal measurements [6, 11].

However, it is unclear if maternal or cord blood vitamin D concentrations are associated with differences in child temperament. Temperament refers to early-emerging, individual differences in behavior that are relatively constant across time, and can predict mental health throughout the lifespan [19, 20]. Thus, an association between 25(OH)D and temperament could also help understand later-emerging differences in mental health.

In the current study, we compared results from two independent well-characterized studies: the Vitamin D Intervention in Infants (VIDI) study from Finland, and the Generation R Study, a population-based pregnancy cohort from the Netherlands. Our objective was to assess if maternal 25(OH)D during early or mid-pregnancy and cord blood 25(OH)D at birth are associated with Negative affectivity. This temperament trait is particularly interesting for two main reasons. Firstly, unlike some of the later-emerging traits, Negative affectivity can be assessed early in infancy, during which it manifests as a tendency to cry and express fear and distress easily, and to recover from distress slowly [19, 20]. Secondly, individuals who display more Negative affectivity in infancy have been shown to have an increased risk of ADHD and ASD, and show more internalizing symptoms in childhood and adolescence [19].

## Methods

### Participants

VIDI originally comprised 975 infants born in Helsinki, Finland in 2013–2014, and has been previously described [21, 22]. Briefly, healthy, term-born, appropriate-for-gestational-age (AGA) infants were randomized to daily vitamin D3 supplementation of 10 μg (standard-dose) or 30 μg (high-dose) from 2 weeks to 2 years of life (ClinicalTrials.com registration NCT01723852). Data collected before and at birth were observational, and infant supplementation was independent of maternal/cord blood 25(OH)D concentrations and background characteristics (please see Supporting information). Of the 975 infants, 777 (80%) had data on maternal or cord blood 25(OH)D, Negative affectivity, and covariates, and were included in the current study.

The Generation R Study is a multiethnic population-based prospective cohort that has been previously described elsewhere [23]. Briefly, pregnant women living in Rotterdam, the Netherlands, with an expected delivery date in 2002–2006 were recruited. This cohort included 3759 term-born, AGA infants whose parents were born in the Netherlands. Of these 3759 infants, 1505 (40%) had data on maternal or cord blood 25(OH)D, Negative affectivity, and covariates, and were included in the current study.

For more details of study protocols and participant flow, please see Supporting Information.

### Maternal and cord blood vitamin D concentrations

In VIDI, 25(OH)D concentrations were analyzed from maternal blood samples collected at 6–27 weeks of gestation (mean = 11.3, SD = 1.9), and from cord blood collected at birth at 37–42 weeks of gestation (mean = 40.2, SD = 1.1). Maternal pregnancy serum 25(OH)D and cord blood plasma 25(OH)D concentrations were analyzed with a fully automated IDS-iSYS immunoassay system with chemiluminescence detection. Intra-assay variations were 7% (pregnancy) and 13% (cord blood). Linear agreement with liquid chromatography in tandem with mass spectrometry was good (*R*^2^ = 0.942).

In Generation R, 25(OH)D concentrations were analyzed from maternal blood samples collected at 18–25 weeks of gestation (mean = 20.5, SD = 1.0) and from cord blood samples collected at birth at 37–42 weeks of gestation (mean = 40.2, SD = 1.2) [25]. Samples were quantified using isotope dilution liquid chromatography-tandem mass spectrometry: the analytical system consisted of a Shimadzu Nexera UPLC coupled to an AbSciex 5500 QTRAP equipped with an APCI source, with inter-assay imprecision < 11%.

25(OH)D reflected sum of 25(OH)D2 and 25(OH)D3 concentrations. Please see Supporting Information for methodological details.

### Infant temperament: negative affectivity

The Infant Behavior Questionnaire, Revised version (IBQ-R) was used in both studies [24]. IBQ-R yields 14 subscale scores, each of which contribute to one of the 3 broad-band scales. Only the Negative affectivity broadband scale was assessed in both studies.

Parents filled out the questionnaire at the mean child age of 11.7 (SD = 0.58 months) months in VIDI and 6.5 (SD = 0.9) months in Generation R. The four subscale scores, Sadness (indicating lower general mood), Distress to Limitations (crying, fussing, expressing distress when faced with limitations), Fear (becoming distressed/startled when exposed to new situations/stimuli), and Recovery from distress (slower recovery from distress/arousal; inverse-coded so that higher scores on all scales reflect increased Negative affectivity) were standardized within the sample (mean = 0, SD = 1). We calculated Negative affectivity scores as the average score across the four subscales: higher scores indicate more negative affectivity. The assessment is described in more detail in Supporting Information.

### Statistical analyses

We conducted all analyses separately in VIDI and in Generation R. In the main analyses, we used separate multivariate linear regression models to examine associations between maternal 25(OH)D concentration during pregnancy or cord blood 25(OH)D concentration at birth, and Negative affectivity.

Child age and sex were included as covariates in the basic model. Potential maternal confounders were selected based on theoretical background, and included in the adjusted model. These were maternal age (years), self-reported education (upper tertiary/lower tertiary/lower), early-pregnancy BMI (kg/m^2^), smoking during pregnancy (yes/no), and season at the time of 25(OH)D measurement (December-February/March–May/June–August/September–November). In sensitivity analyses, we further adjusted for prenatal maternal (1) self-reported depressive symptoms, and (2) thyrotropin concentrations: both covariates were only available in Generation R. Please see Supporting Information for details on covariate selection and data.

We then ran secondary analyses. First, we tested associations between 25(OH)D concentrations and the four subscale scores: sadness, distress to limitations, fear, and rate of falling reactivity.

Second, we categorized 25(OH)D concentration into five groups: < 25 nmol/l, 25–49.9 nmol/l, 50–74.9 nmol/l, 75–125 nmol/l, and > 125 nmol/l. To identify vitamin D insufficiency in the general population, a cut-off of 50 nmol/l is often recommended, however, lower cut-offs (such as < 25 nmol/l [25]) can be used to identify profound vitamin D deficiency [25–28]. Higher cut-offs (such as < 75 nmol/l [25]) have been recommended to identify levels that, despite being sufficient for bone health, may be non-optimal for perinatal, metabolic and other health outcomes [25, 29]. Finally, some studies suggest that concentrations beyond > 125 nmol/l may be associated with harm, and the US Institute of Medicine for example has adopted this cut-off to select tolerable upper intake levels [27, 28]. Because there is no consensus over the optimal categorization of maternal or cord blood 25(OH)D, we used these previously recommended cut-offs to categorize 25(OH)D. In linear regression models, we used the group with 25(OH)D 75–125 nmol/l (“optimal”) as the reference group, and compared participants with 25(OH)D deficiency (< 25 nmol/l), insufficiency (25–49.9 nmol/l), suboptimal concentration (50–74.9 nmol/l), and high concentration (> 125 nmol/l) against this reference group. In VIDI, only one participant had 25(OH)D < 25 nmol/l during pregnancy and was excluded in the categorical analyses. None of the VIDI participants had 25(OH)D < 25 nmol/l at birth. In Generation R, only two participants had 25(OH)D > 125 nmol/l at birth, and were excluded in the categorical analyses.

Third, we examined potential U-shaped associations by adding a quadratic term of 25(OH)D in the main models. Some evidence suggests that both high and low neonatal 25(OH)D concentrations could be associated with poorer mental health outcomes [30].

Fourth, we examined moderation by sex by adding an interaction term (child sex × 25(OH)D concentration) into the main models. Some previous evidence suggests that associations between maternal 25(OH)D and child behavioral symptoms vary by sex [9].

Finally, in non-response analyses, we compared analytical sample against those who were not included in the study because of missing data, using independent t-tests, Mann–Whitney U tests, and chi-squared tests. In Generation R, 22% of otherwise eligible participants were lost due to missing covariate data (compared with only 5% in VIDI). In supplementary analyses, we added these otherwise eligible Generation R participants into the analytical sample, used MICE in R (30 datasets, 10 iterations) to impute missing covariate data, and re-ran the fully adjusted main analyses in Generation R [31].

## Results

**Fig. 1:**
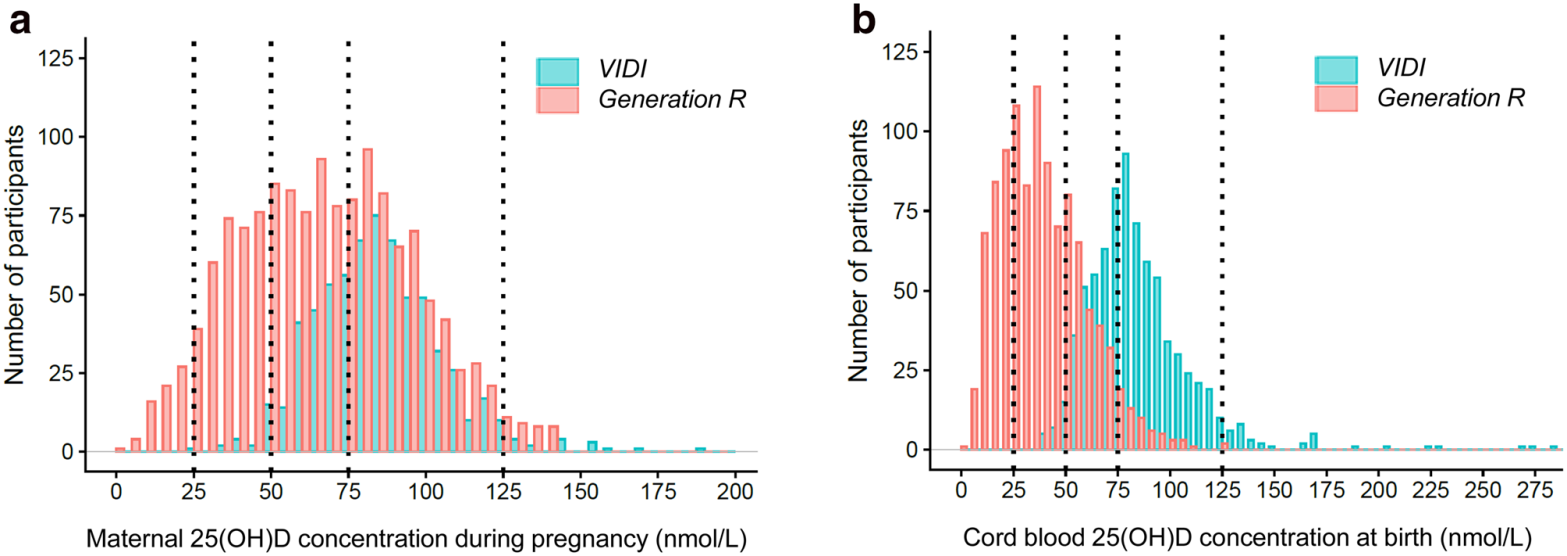
The distribution of maternal 25(OHD) concentrations during pregnancy (**a**) and cord blood 25(OH)D concentrations at birth (**b**)

Table 1 presents the sample characteristics. On average, the 777 mothers in VIDI were 31.2 years old, 74% had BMI 18.5–24.9 kg/m^2^, 14.1% smoked during pregnancy, and 46.6% had upper tertiary education. Among the 1,505 mothers in Generation R, average age was 31.6 years, 66.9% had BMI 18.5–24.9 kg/m^2^, 21.9% smoked during pregnancy, and 45.1% had upper tertiary education. Figure 1 illustrates maternal 25(OH)D concentrations during pregnancy (Fig. 1a) and cord blood 25(OH)D concentrations at birth (Fig. 1b). We report results from the adjusted models in the text (for results from basic models, please see Supplementary Table 1).

**Table 1:** Characteristics of the VIDI and Generation R Study participants

Characteristics	VIDI		Generation R	
	Total *N* = 777		Total *N* = 1505	
	Mean (SD) [range] or *n* (%)	*N*	Mean (SD) [range] or *n* (%)	*N*
Maternal characteristics				
Pregnancy 25(OH)D, nmol/l	82.7 (20.4) [24.8, 189.2]	651	69.3 (28.2) [3.8, 144.6]	1398
< 25 nmol/l, *n* (%)	1 (0.2)		67 (4.8)	
25–49.9 nmol/l, *n* (%)	23 (3.5)		319 (22.8)	
50–74.9 nmol/l, *n* (%)	209 (32.1)		418 (27.8)	
75–125 nmol/l, *n* (%)	402 (61.8)		558 (37.1)	
> 125 nmol/l, *n* (%)	16 (2.5)		36 (2.4)	
Pregnancy 25(OH)D measurement season		651		1398
Winter (Dec, Jan, Feb), *n* (%)	144 (22.1)		290 (20.7)	
Spring (Mar, Apr, May), *n* (%)	97 (14.9)		372 (26.6)	
Summer (Jun, Jul, Aug), *n* (%)	168 (25.8)		403 (28.8)	
Autumn (Sep, Oct, Nov), *n* (%)	242 (37.2)		333 (23.8)	
GA at 25(OH)D measurement, weeks	11.3 (1.9) [6.1, 27.1]	651	20.5 (1.0) [18.1, 24.8]	1398
Age at enrolment, years	31.2 (4.3) [19.0, 44.0]	777	31.6 (4.2) [16.8, 46.3]	1505
Early-pregnancy BMI, kg/m^2^		777		1505
< 18.5, *n* (%)	21 (2.7)		29 (1.9)	
18.5–24.9, *n* (%)	575 (74.0)		1007 (66.9)	
25–29.9, *n* (%)	135 (17.4)		339 (22.5)	
30 or more, *n* (%)	46 (5.9)		130 (8.6)	
Smoking, *n* (%)	109 (14.1)	777	330 (21.9)	1505
Educational level		777		1505
Primary/secondary, *n* (%)	189 (24.3)		407 (27.0)	
Lower tertiary, *n* (%)	226 (29.1)		419 (27.8)	
Upper tertiary, *n* (%)	362 (46.6)		679 (45.1)	
BSI depression score during pregnancy	NA		0.11 (0.32) [0, 3.7]	1374
BSI depression score > 0.8, *n* (%)	NA		53 (3.9)	
Thyrotropin concentration, mU/l	NA		1.7 (1.4) [0.0, 25.4]	1137
Infant characteristics				
Cord blood 25(OH)D, nmol/l	82.8 (26.3) [36.7, 283.7]	763	40.6 (20.5) [4.9, 129.5]	1053
< 25 nmol/l, *n* (%)	0 (0)		265 (25.2)	
25–49.9 nmol/l, *n* (%)	27 (3.5)		466 (44.3)	
50–74.9 nmol/l, *n* (%)	287 (37.6)		259 (24.6)	
75–125 nmol/l, *n* (%)	415 (54.4)		61 (5.8)	
> 125 nmol/l, *n* (%)	34 (4.5)		2 (0.2)	
Cord blood 25(OH)D measurement season		763		1053
Winter (Dec, Jan, Feb), *n* (%)	144 (18.9)		298 (28.3)	
Spring (Mar, Apr, May), *n* (%)	322 (42.2)		267 (25.4)	
Summer (Jun, Jul, Aug), *n* (%)	168 (22.0)		275 (26.1)	
Autumn (Sep, Oct, Nov), *n* (%)	129 (16.9)		213 (20.2)	
Sex, female, *n* (%)	498 (51.2)	777	751 (49.9)	1505
Age at IBQ-R assessment, months	11.7 (0.58) [8.9, 16.1]	777	6.5 (0.9) [4.7, 11.9]	1505

Data are means (SD) [range] unless stated otherwise. 25(OH)D concentration categorization was based on previously recommended cut-offs for 25(OH)D deficiency (< 25 nmol/l), insufficiency (< 50 nmol/l), suboptimal concentration (< 75 nmol/l), and high concentration (> 125 nmol/l) [25–27]

*25(OH)D* 25-hydroxyvitamin D, *BSI* Brief symptom inventory, *BMI* body-mass-index, *GA* gestational age, *IBQ-R* Infant Behavior Questionnaire, Revised, *N* number of participants with available data, *n* number of cases, *NA* Data not available, *SD* standard deviation, *%* proportion of cases among those with data available

Mothers in the VIDI analytical sample were less often smokers and had higher 25(OH)D in pregnancy, and their offspring had higher cord blood 25(OH)D at birth and were younger at follow-up, compared with those who could not be included in the analytical sample (*n* = 198, Supplementary Table 2). In Generation R, mothers in the analytical sample were older, more highly educated, less often depressed, less often smoked, and had higher 25(OH)D concentrations during pregnancy, and the infants in the analytical sample were younger, compared with those who could not be included (*n* = 2254) (Supplementary Table 2).

### Maternal 25(OH)D during pregnancy and infant temperament

In both cohorts, higher maternal 25(OH)D concentration during pregnancy was associated with lower infant Negative affectivity, when controlling for child sex and age, and maternal age, education, BMI, smoking, and season of 25(OH)D measurement (Table 2). The effect estimates were similar across cohorts: for every 10 nmol/l increase in maternal 25(OH)D, infants had 0.03 SD units (95% CI − 0.06 to − 0.004) and 0.02 SD units (95% CI − 0.03 to − 0.01) lower Negative affectivity scores in VIDI and Generation R, respectively (Table 2). This association remained similar after additionally adjusting for maternal depressive symptoms (− 0.02 SD, 95% CI − 0.03 to − 0.01) or thyrotropin (− 0.03 SD, 95% CI − 0.04, − 0.01) (depression and thyrotropin data only available in Generation R). In subscale analyses, lower maternal 25(OH)D was associated with higher distress to limitations and slower recovery from distress in both cohorts, and with higher sadness in Generation R (Supplementary Table 3).

**Table 2:** Associations between 25(OH)D concentrations measured during pregnancy and at birth and infant negative affectivity in VIDI and in the Generation R Study

	EE (95% CI)	*p*
Maternal 25(OH)D in pregnancy		
VIDI	− 0.03 (− 0.06, − 0.004)	0.02
Generation R Study	− 0.02 (− 0.03, − 0.01)	0.01
Cord blood 25(OH)D at birth		
VIDI	0.00 (− 0.02, 0.02)	0.92
Generation R Study	− 0.03 (− 0.05, − 0.01)	0.01

Effect estimates are presented as change in Negative Affectivity, in SD units, per each 10 nmol/l increase in 25(OH)D concentration. Higher Negative affectivity reflects a tendency to cry and express fear and distress more easily and to recover from distress more slowly. We adjusted for child sex, age at assessment, maternal age, educational level, early-pregnancy BMI, smoking, and season of 25(OH)D measurement

*25(OH)D* 25-hydroxyvitamin D, *EE* non-standardized effect estimate from linear regression model, *CI* confidence interval for effect estimate, *p* *p* value

Table 3 shows associations between categorical maternal 25(OH)D in pregnancy and infant Negative affectivity. In both samples, the infants whose mothers had the lowest 25(OH)D during pregnancy had the highest Negative affectivity in infancy, but most differences between the groups were not statistically significant. Only Generation R had enough mothers with 25(OH)D < 25 nmol/l during pregnancy to test if vitamin D deficiency, defined using this strict cut-off, was associated with infant temperament: compared with infants whose mothers had “optimal” 25(OH)D (75–125 nmol/l), the infants whose mothers had 25(OH)D < 25 nmol/l had 0.25 SD units higher Negative affectivity (95% CI 0.08–0.42) (Table 3). Infants whose mothers had high 25(OH)D (> 125 nmol/l) did not differ from the reference group (75–125 nmol/l) (Table 3).

**Table 3:** Associations between 25(OH)D concentrations measured during pregnancy and at birth and categorized into five distinct categories, and infant negative affectivity

	*N*	EE (95% CI)	*p*
Maternal 25(OH)D in pregnancy			
VIDI			
< 25 nmol/l	1	n/a	
25–49.9 nmol/l	23	0.29 (− 0.01, 0.59)	0.06
50–74.9 nmol/l	209	0.11 (− 0.02, 0.23)	0.09
75–125 nmol/l	402	Ref	
> 125 nmol/l	16	− 0.05 (− 0.41, 0.31)	0.78
Generation R Study			
< 25 nmol/l	67	0.25 (0.07, 0.42)	0.01
25–49.9 nmol/l	319	0.09 (− 0.01, 0.19)	0.06
50–74.9 nmol/l	418	− 0.01 (− 0.10, 0.08)	0.82
75–125 nmol/l	558	Ref	
> 125 nmol/l	36	− 0.15 (− 0.38, 0.08)	0.21
Cord blood 25(OH)D at birth			
VIDI			
< 25 nmol/l	0	n/a	
25–49.9 nmol/l	27	0.01 (− 0.29, 0.30)	0.97
50–74.9 nmol/l	287	0.01 (− 0.10, 0.12)	0.87
75–125 nmol/l	415	Ref	
> 125 nmol/l	34	− 0.05 (− 0.31, 0.21)	0.72
Generation R Study			
< 25 nmol/l	265	0.04 (− 0.43, 0.52)	0.85
25–49.9 nmol/l	466	0.01 (− 0.36, 0.37)	0.98
50–74.9 nmol/l	259	0.12 (− 0.14, 0.38)	0.36
75–125 nmol/l	61	Ref	
> 125 nmol/l	2	n/a	

Effect estimates are presented as change in Negative Affectivity, in SD units, from linear regression models where each of the groups was compared against the reference group with 25(OH)D concentration between 75 and 125 nmol/l. Higher Negative affectivity reflects a tendency to cry and express fear and distress more easily and to recover from distress more slowly. We adjusted for child sex, age at assessment, maternal age, educational level, early-pregnancy BMI, smoking, and season of 25(OH)D measurement

*25(OH)D* 25-hydroxyvitamin D, *EE* non-standardized effect estimate from linear regression model, *CI* confidence interval for effect estimate, *n/a* not applicable, this model could not be run because of the small number of cases, *p* *p* value, *ref* reference group

Inclusion of a quadratic term in the models did not suggest a U-shaped association between maternal 25(OH)D and infant Negative affectivity (*p* values > 0.09).

### Cord blood 25(OH)D at birth and temperament in infancy

In VIDI, we observed no association between cord blood 25(OH)D concentrations at birth and Negative affectivity (Table 2). In Generation R, however, higher 25(OH)D at birth was associated with lower infant Negative affectivity: per 10 nmol/l increase in cord blood 25(OH)D concentration at birth, negative affectivity scores were 0.03 SD lower in infancy (95% CI − 0.05, − 0.01) (Table 2). This association remained similar when we additionally adjusted for maternal depressive symptoms (− 0.04, 95% CI − 0.06, − 0.01) or thyrotropin (− 0.03, 95% CI − 0.06, − 0.003). When examining subscale-level associations, higher cord blood 25(OH)D was associated with lower distress to limitations and faster recovery from distress (in Generation R) (Supplementary Table 3).

However, when we compared infants with cord blood 25(OH)D < 25 nmol/l, 25–50 nmol/l, 50–75 nmol/l, or > 125 nmol/l, to the reference group (75–125 nmol/l), we found no differences in Negative affectivity in either cohort (Table 3). Table 3 also illustrates the lack of any clear pattern of effects (where the groups with the lowest cord blood 25(OH)D concentrations would have the highest Negative affectivity), in contrast to the pattern observed for maternal 25(OH)D during pregnancy.

Finally, we found no evidence of a U-shaped association between cord blood 25(OH)D at birth and Negative affectivity in either cohort (quadratic term *p* values > 0.58).

### Moderation by sex

There was no evidence of an interaction between infant sex and maternal or cord blood 25(OH)D concentration in predicting infant Negative Affectivity in either cohort (*p* values > 0.21).

### Non-response analyses

After imputing missing covariates in the otherwise eligible Generation R sample (*n* = 1930) (Supplementary Table 2), associations between 25(OH)D in pregnancy and at birth and Negative affectivity remained very similar compared with the results from non-imputed data (per 10 nmol/l increase in maternal 25(OH)D, Negative affectivity was − 0.02 SD units lower [*p* = 0.001]; per 10 nmol/l increase in cord blood 25(OH)D, Negative affectivity was − 0.03 SD units lower [*p* = 0.01]).

## Discussion

The present study demonstrated that higher maternal vitamin D concentration during pregnancy was associated with lower offspring Negative affectivity in infancy. Infants whose mothers had higher 25(OH)D concentration in early- and mid-pregnancy displayed lower distress to limitations and better recovery from distress at 6–12 months of age. This finding was similar across two independent samples of mother–child dyads from Finland and from the Netherlands.

We did not find evidence of a U-shaped association. Infants whose mothers had 25(OH)D concentrations above 125 nmol/l had similar levels of Negative affectivity, compared with those who had concentrations between 75 and 125 nmol/l. However, any adverse effects of extremely high 25(OH)D were beyond the scope of our study.

Somewhat surprisingly, vitamin D concentration at birth, measured from cord blood, was not consistently associated with infant Negative affectivity. In VIDI, we observed no association between cord blood 25(OH)D and Negative affectivity. In Generation R, lower cord blood 25(OH)D was associated with higher Negative affectivity when modelling 25(OH)D as a continuous variable. However, even within Generation R, the pattern of findings for categorized cord blood 25(OH)D was not as clear as the pattern observed for maternal 25(OH)D: infants with the lowest cord blood 25(OH)D concentrations did not have the highest Negative affectivity. We are not aware of any previous studies on cord blood 25(OH)D and temperament, and thus advise caution in interpreting these mixed results for cord blood. While it is possible that low 25(OH)D at birth is associated with higher Negative affectivity, the association in the current study was not as consistent as that between maternal early/mid-pregnancy 25(OH)D and Negative affectivity.

Some indirect prior evidence supports the interpretation that early- and mid-pregnancy vitamin D concentrations could be important for fetal brain development, which in turn could help explain the temperamental differences observed in the current study. During this early period, fundamental structural development of the brain, including regions that are vital for behavioral regulation such as the prefrontal and limbic regions, occurs [32, 33]. In animal studies, vitamin D concentrations have been linked with multiple processes that take place during early fetal brain development, including neuronal migration and development of dendritic morphology and neuronal connectivity [6, 34]. A recent meta-analysis suggested that early- to mid-gestation 25(OH)D concentrations could have a stronger effect on the neurodevelopment of the offspring, compared with 25(OH)D concentrations later in gestation [11].

The difference in Negative effectivity was approximately one-quarter of a standard deviation, between infants whose mothers had lowest (< 25 nmol/l) vs “optimal” (75–125 nmol/l) 25(OH)D concentrations during pregnancy. Such small differences in an infant’s tendency to express distress are not directly relevant for guiding clinical decision-making. However, identifying even subtle population-level effects can be meaningful, as understanding early environmental factors that influence behavioral phenotypes can elucidate the etiology of mental disorders, and help identify vulnerable populations and modifiable environmental risk factors. While we are not aware of previous studies on prenatal 25(OH)D concentrations and infant temperament, associations between prenatal vitamin D concentrations and psychopathology have been observed previously [9–14]. Negative affectivity, in turn, has been associated with psychopathology, including increased risk of ADHD, ASD, and internalizing in childhood and adolescence. [19] We encourage research into temperament as a potential mediating factor between maternal 25(OH)D during pregnancy and child psychopathology.

The current study has several strengths. First, a similar association between maternal 25(OH)D and infant Negative affectivity was shown in two independent samples, reducing the risk of chance findings and suggesting generalizability beyond just one country or population. Secondly, we used a standardized instrument, IBQ-R, to assess temperament in both samples. Thirdly, we addressed several potential sources of confounding by controlling for maternal age, education, smoking, BMI, depressive symptoms, thyrotropin, and season of 25(OH)D measurement. Further, to address potential confounding by ethnicity, we analyzed study samples that were ethnically homogenous. Finally, we measured 25(OH)D both in early/mid-pregnancy and at birth.

Some limitations need to be acknowledged. The low prevalence of low 25(OH)D (< 25 nmol/l) in VIDI and of high 25(OH)D (> 125 nmol/l) in Generation R, especially at birth, limited the direct comparison of some results between the two cohorts, and could limit power to detect effects and e.g., cause underestimation of the effect magnitude. Two laboratories independently assessed 25(OH)D concentrations, and while differences in 25(OH)D status are likely to at least partly reflect true differences between populations [35, 36], we cannot rule out these were influenced by some methodological differences. Observed 25(OH)D concentrations may also not perfectly capture the bioavailable amount of vitamin D, potentially introducing imprecision. Moreover, attrition can further limit generalizability to families with lower socioeconomic status. Further, any generalized conclusions based on these two studies in high-income European countries should be drawn with caution, as the effects could vary by population [6, 37]. All infants in VIDI received standard or high-dose vitamin D supplementation, and Dutch guidelines also recommended vitamin D supplementation for infants, limiting the number of children exposed to both maternal and infant vitamin D insufficiency and any potentially additive effects [38]. Altogether, postnatal factors that could mediate or moderate associations between maternal and fetal vitamin D status and infant temperament were mostly beyond the scope of this study. While more research is needed to identify such factors, the fact that we observed these associations among AGA, term-born infants suggests that key perinatal complications such as preterm birth could not fully explain them. Finally, while several potential confounders were addressed, the risk of residual confounding inevitably remains (e.g., by genetic factors, maternal morbidities, and lifestyle factors explaining differences in nutritional status).

## Conclusion

The current study showed an association between higher maternal vitamin D status during pregnancy and lower infant Negative affectivity. This supports the hypothesis that vitamin D plays a role in fetal neurodevelopment and is associated with early-emerging differences in child behavior.

## Supplementary Information

Below is the link to the electronic supplementary material.

Supplementary file1 (DOCX 47 KB)

Supplementary file2 (DOCX 42 KB)

## Data Availability

Requests to access VIDI and Generation R Study data can be sent to the corresponding author Kati Heinonen (kati.heinonen-tuomaala@tuni.fi) or to the Generation R Study data management (generationr@erasmusmc.nl), respectively. Requests to access individual-level data are subject to assessment and approval by the management teams of the studies, to ensure the privacy of the participants, which is protected by law.

## Code availability

Codes are available on request from the corresponding author Kati Heinonen (kati.heinonen@tuni.fi).
