# Supplementary material for: Prenatal maternal and cord blood vitamin D concentrations and negative affectivity in infancy
Source: Eur Child Adolesc Psychiatry. 2021 Oct 18;32(4):601–9. doi: 10.1007/s00787-021-01894-4 (PMC10115713; doi:10.1007/s00787-021-01894-4)
Supplement: Supplementary file 1 — Supplementary file1 (DOCX 47 KB) [file 787_2021_1894_MOESM1_ESM.docx]

**Prenatal maternal and cord blood vitamin D concentrations and negative affectivity in infancy**

**European Child & Adolescent Psychiatry**

Sara Sammallahti, Elisa Holmlund-Suila, Runyu Zou, Saara Valkama, Jenni Rosendahl, Maria Enlund-Cerullo, Helena Hauta-alus, Marius Lahti-Pulkkinen, Hanan El Marroun, Henning Tiemeier, Outi Mäkitie, Sture Andersson, Katri Räikkönen, Kati Heinonen

Corresponding author: Kati Heinonen, [kati.heinonen-tuomaala@tuni.fi](mailto:kati.heinonen-tuomaala@tuni.fi)

**Supporting information: Methods Supplement**

[1. Details of the participants of the VIDI study 2](#_Toc72861303)

[2. Details of the participants of the Generation R Study 4](#_Toc72861304)

[3. Details of the maternal and cord blood vitamin D concentration measurements 5](#_Toc72861305)

[4. Details of the assessments of Negative affectivity 7](#_Toc72861306)

[5. Details of the covariate data 9](#_Toc72861307)

[6. Funding for the VIDI and Generation R Study 11](#_Toc72861308)

[References 12](#_Toc72861309)

# 1. Details of the participants of the VIDI study

VIDI is a randomized controlled trial that originally comprised 975 healthy term infants. These infants were randomized to daily vitamin D3 supplementation of either 10 μg or 30 μg from 2 weeks to two years of life. The study protocol has been described in detail previously(Helve et al., 2017; Rosendahl et al., 2018). Briefly, mother-infant dyads were recruited at the Kätilöopisto Maternity hospital in Helsinki, Finland immediately (within ~2 days) after delivery between January 2013 and June 2014.

All participating mothers were of Northern European ethnicity and used no regular medication. The infants were singletons, born at term (37 to 42 weeks of gestation) with a birth weight appropriate for gestational age (AGA; birth weight standard deviation [SD] score between −2.0 to +2.0 (Pihkala, Hakala, Voutilainen, & Raivio, 1989)). Further exclusion criteria for the study were nasal continuous positive airway pressure treatment for more than 1 day, intravenous glucose infusion, intravenous postnatal antibiotic treatment, seizures, duration of phototherapy for more than 3 days, and need for nasogastric tube for more than 1 day.

The study was approved by the Research Ethics Committee of the Hospital District of Helsinki and Uusimaa (107/13/03/03/ 2012), conducted according to the Declaration of Helsinki, and registered into ClinicalTrials.com (NCT01723852). Written informed consent was obtained from all mothers at recruitment.

Of the 975 mother-infant dyads who were originally recruited into VIDI, 970 had 25(OH)D data available. These 25(OH)D concentrations were analyzed from maternal serum samples collected during the first two trimesters of pregnancy (n=808) or from umbilical cord blood samples collected at birth (n= 955) (see “Maternal and cord blood vitamin D concentrations”). Of these 970 infants, 823 had data on temperament rated by their parents (see “Infant temperament: Negative affectivity”). After further excluding those with missing covariate data (see Model II in “Statistical analyses”, n=46), the final analytical sample included 777 participants (80% of the original sample): 651 had 25(OH)D data during pregnancy, and 763 at birth.

Randomization of infants to vitamin D3 supplementation with either the standard recommend dose of 10 μg per day, or with the higher dose of 30 μg per day was performed after the collection of observational pre- and perinatal data. Supplementation status was independent of these earlier data, ensuring routine vs high-dose supplementation would neither dilute nor mediate the effects of prenatal factors on child development. Detailed comparisons showing the similarity of the children in the two treatment groups at baseline (randomization at 2 weeks of age) have been previously published (Rosendahl et al., 2018). Also in the current analytical sample of 777 mother-child dyads, those who were randomized to receive high-dose vitamin D supplementation (n=391) vs standard-dose vitamin D supplementation (n=386) in infancy did not differ in maternal 25(OH)D concentration during pregnancy (25(OH)D: M=82.6, SD=18.0 in high-dose group, M=82.8, SD=22.5 in standard dose group, p=0.89) or in cord blood 25(OH)D concentration at birth (25(OH)D: M=82.2, SD=23.7 in high-dose group, M=83.4, SD=28.6 in standard dose group, p=0.56), when comparing the two groups using t-tests.

# 2. Details of the participants of the Generation R Study

The Generation R Study is a population-based prospective cohort that has been previously described elsewhere (Jaddoe et al., 2006). Briefly, all pregnant women living within clearly defined areas of Rotterdam, the Netherlands, with an expected delivery date between April 2002 and January 2006, were invited to participate.

The study was approved by the Medical Ethical Committee of the Erasmus Medical Centre in Rotterdam and conducted according to the Declaration of Helsinki. Written informed consent was obtained from all participating mothers.

Of the 8,976 mothers originally enrolled in the multi-ethnic Generation R Study during pregnancy, we only included mothers who were of Dutch national origin (n=4,477), to overcome issues related to ethnic differences in vitamin D metabolism and distribution (Holick, 1995). The mother was considered Dutch if both her parents had been born in the Netherlands, according the classification of Statistics Netherland (Jaddoe et al., 2006). We further excluded non-live births and neonatal deaths (n=62), non-singletons (n=126), and children who were non-AGA, non-term-born, or both (n=445) (i.e. all included infants had birth weight −2.0 to +2.0 and gestational age 37 to 42 weeks of gestation). Finally, we randomly excluded one child per sibling pair (n=85). Of the remaining 3,759 infants who were eligible for the current study, 3,488 had 25(OH)D data available (n=3,160 had data on maternal 25(OH)D during pregnancy and n=2,439 had data on cord blood 25(OH)D at birth). Of these infants, 1,930 had data on temperament rated by their caregivers. After further excluding those with missing covariate data in all primary analyses (n=425), the final analytical sample included 1,505 participants (40% of eligible sample) (n=1,398 had 25(OH)D data during pregnancy and n=1,053 at birth).

# 3. Details of the maternal and cord blood vitamin D concentration measurements

In VIDI, maternal serum samples during pregnancy were collected as part of mothers’ routine follow-up visit at prenatal clinics at 6-27 weeks of gestation (mean=11.3, SD=1.9). Pregnancy samples were stored as part of the national maternity care plan in the Finnish Maternity Cohort serum bank and acquired from there for use in the current study. Cord blood samples were collected at birth at 37-42 weeks of gestation (mean=40.2, SD=1.1). Maternal pregnancy serum 25(OH)D and cord blood plasma 25(OH)D concentrations were analyzed with a fully automated IDS-iSYS immunoassay system with chemiluminescence detection (Immunodiagnostic Systems Ltd., Bolton, United Kingdom). Intra-assay variations were 7% for pregnancy 25(OH)D, and 13% for cord blood 25(OH)D. The method shows good linear agreement with liquid chromatography in tandem with mass spectrometry (LC-MS, R2=0.942, in-house comparison performed with 67 samples) (Helve et al., 2017). The mean (95% CI) value for the ratio of IDS-iSYS 25(OH)D to LC-MS 25(OH)D concentration was 0.73 (0.68; 0.78) (Helve et al., 2017). Detailed information can be found elsewhere (Helve et al., 2017; Rosendahl et al., 2018). We identified and truncated outliers (maternal values >150 nmol/L during pregnancy were encoded to 150 nmol/ L, n=6; and cord blood values >170 nmol/L at birth were encoded to 170 nmol/L, n=7) using the Mahalanobis method (p<0.001). We truncated rather than trimmed outliers, because we recognized the excessive influence these values could have in the statistical models, however we had no reason to assume they represented measurement error.

In Generation R, 25(OH)D concentrations were analyzed from maternal blood samples collected at 18-25 weeks of gestation (mean=20.5, SD=1.0) and from cord blood samples collected at birth at 37-42 weeks of gestation (mean=40.2, SD=1.2) (Vinkhuyzen et al., 2018). Samples were quantified using isotope dilution liquid chromatography-tandem mass spectrometry: the analytical system consisted of a Shimadzu Nexera UPLC coupled to an AbSciex 5500 QTRAP equipped with an APCI source (Vinkhuyzen et al., 2018). Linearity of 25(OH)D concentration was assessed using matrix-matched calibration standards, with R2 values of >0.99 across the calibration range (10 – 125 nmol/L). Assay accuracy was assessed at four concentration levels for 25(OH)D3 (48.3, 49.4, 76.4, 139.2 nmol/L) and a single level for 25(OH)D2 (32.3 nmol/L) using certified reference materials purchased from the National Institute of Standards and Technology (NIST) (NIST SRM 972a Levels 1-4), and was excellent at all concentration levels tested (<10% and <17%, respectively) (Vinkhuyzen et al., 2018). Assay repeatability was assessed via replicate analysis of an independent reference material (NIST SRM1950, 61.9 nmol/L 25OHD3), and inter-assay imprecision was <11% (n=343) (Vinkhuyzen et al., 2018). The 25(OH)D values were approximately normally distributed in the Generation R Study and we observed no extreme outliers.

# 4. Details of the assessments of Negative affectivity

The Infant Behavior Questionnaire, Revised version (IBQ-R) was used in both studies (Gartstein & Rothbart, 2003). IBQ-R includes 191 items. Parents were asked to rate how often their child has behaved in a described way in everyday situations (range 1 = Never to 7 = Always).

The IBQ-R yields 14 subscale scores, each of which contribute to one of the 3 broad-band scales. Only the Negative affectivity broadband scale was assessed in both samples. This scale was composed of four subscales. Higher scores on the Sadness (indicating lower general mood, e.g. appearing sad when caregiver has been away unusually long), Distress to Limitations (crying, fussing and showing distress when faced with limitations, e.g. during caretaking activities), and Fear subscales (becoming distressed or startled when exposed to new or unusual situations and stimuli, e.g. when hearing a loud noise) contribute to higher Negative affectivity. Lower scores on the Recovery from distress subscale (indicating slower recovery from distress or arousal, e.g. calming down slowly after crying) contribute to higher Negative affectivity (Gartstein & Rothbart, 2003).

In both studies, we calculated subscale scores by dividing the sum of item scores by the number of completed items. The Recovery from distress subscale score was reverse-coded (so that higher scores reflect more Negative affectivity on all four subscales). We standardized all subscale scores within the sample (mean=0, SD=1) to facilitate the comparison of results across the two study samples. Finally, Negative affectivity scores were calculated as the average score across the four subscales (i.e. by dividing the sum of the standardized subscale scores by the number of subscales).

In VIDI, a parent filled out the full version of the IBQ-R at the 12-month follow-up (mean age 11.7 months, SD=0.58 months). Internal consistencies (Chronbach’s alphas) of Negative affectivity subscales ranged from 0.72 to 0.83. The internal consistency of the Negative affectivity broadband scale (i.e. across 4 subscale scores) was 0.68.

In Generation R, a parent filled out the IBQ-R at the 6-month follow-up (mean age 6.5 months, SD=0.9) (Roza et al., 2008). The questionnaire was abbreviated for the purpose of feasibility: out of the three broadband scales, only Negative affectivity was assessed. Further, based on a pilot study, a few items deemed as overlapping were removed and the original 7-point scale was transformed into a 3-point scale (Roza et al., 2008). Internal consistencies of the four adapted IBQ-R subscales ranged between 0.75 and 0.86 and were thus comparable to those of the original IBQ-R (Gartstein & Rothbart, 2003). The internal consistency of the Negative affectivity scale was 0.73.

# 5. Details of the covariate data

We included the sex of the child and age at temperament assessment as covariates in all analyses.

In the adjusted models, we added potential confounders, identified based on previous literature (Bornstein et al., 2015; Forrest & Stuhldreher, 2011; Spry et al., 2020; Vinkhuyzen et al., 2016). These included maternal age at enrolment, education, body-mass-index (BMI), and smoking, and season of 25(OH)D measurement.

Maternal education was self-reported during pregnancy and categorized into primary/secondary, vs lower tertiary, vs upper tertiary.

Early-pregnancy body-mass-index (BMI, kg/m2) was assessed at approximately 8 weeks of pregnancy in VIDI, and at approximately 14 weeks in Generation R.

In VIDI, mothers self-reported if they had smoked before pregnancy or not. In Generation R, mothers prospectively reported if they smoked during pregnancy. In both cohorts, maternal smoking was then categorized into yes/no.

Season at the time of venipuncture during pregnancy and of birth was categorized into winter (Dec, Jan, Feb), spring (Mar, Apr, May), summer (Jun, Jul, Aug) or autumn (Sep, Oct, Nov) to take into account the variation in daylight hours and 25(OH)D concentrations (Hauta-alus et al., 2018).

In sensitivity analyses, we examined potential confounding by 1) maternal depressive symptoms and 2) maternal thyroid function, both measured during early- or mid-pregnancy. These two factors could be associated with maternal vitamin D status and child behavioural development, but the causal relationships underlying these associations remain unclear.(Ge et al., 2020; Parker, Brotchie, & Graham, 2017; Stein et al., 2014; Taheriniya, Arab, Hadi, Fael, & Askari, 2021) Data on these covariates were only available in Generation R:

1. In Generation R, maternal depressive symptoms were assessed in mid-pregnancy in a subgroup of the analytical sample (n=1,374, 91.3%; mean gestational age 20.4, SD=1.0, range 18-25 weeks) using the 6-item depression scale of the Brief Symptom Inventory self-report (Cents et al., 2013; de Beurs, 2009). Mothers with a score above 0.8 were considered to have clinically relevant depressive symptoms, and this dichotomous covariate was added in the fully adjusted model in a supplementary sensitivity analysis (Cents et al., 2013; de Beurs, 2009).
2. In Generation R, maternal thyrotropin (i.e., thyroid-stimulating hormone) concentrations, measured from blood samples taken at <18 weeks of pregnancy, were available for a total of 1,137 participants in the current study (mean thyrotropin 1.7 mU/L, SD=1.4, all measurements made prior to measurement of maternal vitamin D measurements). (Henrichs et al., 2010) Thyrotropin concentration was entered as a continuous covariate into the fully adjusted model in a supplementary sensitivity analysis.

# 6. Funding for the VIDI and Generation R Study

The authors have no financial interests or potential conflicts of interest to declare.

The general design of the Generation R Study was supported by the Netherlands Organization for Scientific Research (NWO) and the Dutch Ministry of Health, Welfare, and Sport. The work of the authors involved in the Generation R Study in this project was supported by the LEaDing Fellows EU Marie Skłodowska-Curie COFUND Programme and the Orion Research Foundation (Dr Sammallahti); NWO Vici Grant 016.VICI.170.200 (Dr Tiemeier); and Stichting Volksbond Rotterdam, the Brain & Behavior Research Foundation NARSAD Young Investigator Grant 27853, and the European Union's Horizon 2020 Research and Innovation Program LifeCycle grant No. 733206 (Dr El Marroun). The work of the authors involved in the VIDI study in this project was supported by the Academy of Finland (Dr Heinonen [grant n:o 345057]; Dr Mäkitie, and Dr Lahti-Pulkkinen), by the Sigrid Jusélius Foundation and the Novo Nordisk Foundation (Dr Mäkitie), by the Päivikki and Sakari Sohlberg Foundation and the Juho Vainio Foundation (Dr Hauta-alus), by the Victoriastiftelsen, the Instrumentarium Science Foundation, the Paulo Foundation, and the Orion Research Foundation (Dr Enlund-Cerullo), and by Grants from Special Governmental Subsidy to Clinical Research, the Foundation for Pediatric Research in Finland, and Finska Läkaresällskapet (Dr Andersson).

Funding sources had no role in study design, execution, interpretation or reporting.

# References

Bornstein, M. H., Putnick, D. L., Gartstein, M. A., Hahn, C. S., Auestad, N., & O’Connor, D. L. (2015). Infant Temperament: Stability by Age, Gender, Birth Order, Term Status, and Socioeconomic Status. *Child Development*, *86*(3), 844–863. https://doi.org/10.1111/cdev.12367

Cents, R. A. M., Diamantopoulou, S., Hudziak, J. J., Jaddoe, V. W. V., Hofman, A., Verhulst, F. C., … Tiemeier, H. (2013). Trajectories of maternal depressive symptoms predict child problem behaviour: The Generation R Study. *Psychological Medicine*, *43*(1), 13–25. https://doi.org/10.1017/S0033291712000657

de Beurs, E. (2009). *Brief Symptom Inventory, handleiding addendum [Dutch manual addendum].* Leiden, The Netherlands.

Forrest, K. Y. Z., & Stuhldreher, W. L. (2011). Prevalence and correlates of vitamin D deficiency in US adults. *Nutrition Research*, *31*(1), 48–54. https://doi.org/10.1016/j.nutres.2010.12.001

Gartstein, M. A., & Rothbart, M. K. (2003). Studying infant temperament via the Revised Infant Behavior Questionnaire. *Infant Behavior and Development*, *26*, 64–86.

Ge, G. M., Leung, M. T. Y., Man, K. K. C., Leung, W. C., Ip, P., Li, G. H. Y., … Cheung, C. L. (2020). Maternal thyroid dysfunction during pregnancy and the risk of adverse outcomes in the offspring: A systematic review and meta-analysis. *Journal of Clinical Endocrinology and Metabolism*, *105*(12), 3821–3841. https://doi.org/10.1210/clinem/dgaa555

Hauta-alus, H. H., Holmlund-Suila, E. M., Rita, H. J., Enlund-Cerullo, M., Rosendahl, J., Valkama, S. M., … Viljakainen, H. T. (2018). Season, dietary factors, and physical activity modify 25-hydroxyvitamin D concentration during pregnancy. *Eur J Nutr*, *57*(4), 1369–1379. https://doi.org/10.1007/s00394-017-1417-z

Helve, O., Viljakainen, H., Holmlund-Suila, E., Rosendahl, J., Hauta-alus, H., Enlund-cerullo, M., … Mäkitie, O. (2017). Towards evidence-based vitamin D supplementation in infants: vitamin D intervention in infants (VIDI) — study design and methods of a randomised controlled double-blinded intervention study. *BMC Pediatrics*, *17*, 91.

Henrichs, J., Bongers-Schokking, J. J., Schenk, J. J., Ghassabian, A., Schmidt, H. G., Visser, T. J., … Tiemeier, H. (2010). Maternal thyroid function during early pregnancy and cognitive functioning in early childhood: The generation R study. *Journal of Clinical Endocrinology and Metabolism*, *95*(9), 4227–4234.

Holick, F. (1995). Environmental factors that influence the cutaneous production of vitamin D 1-3. *American Journal of Clinical Nutrition*, *61*, 638S-45S.

Jaddoe, V. W. V, Mackenbach, J. P., Moll, H. A., Steegers, E. A. P., Tiemeier, H., Verhulst, F. C., … Hofman, A. (2006). The Generation R Study: Design and cohort profile. *European Journal of Epidemiology*, *21*(6), 475–484.

Parker, G. B., Brotchie, H., & Graham, R. K. (2017). Vitamin D and depression. *Journal of Affective Disorders*, *208*(October 2016), 56–61. https://doi.org/10.1016/j.jad.2016.08.082

Pihkala, J., Hakala, T., Voutilainen, P., & Raivio, K. (1989). Characteristic of recent fetal growth curves in Finland. *Duodecim*, *105*(18), 1540–1546.

Rosendahl, J., Valkama, S., Holmlund-Suila, E., Enlund-Cerullo, M., Hauta-alus, H., Helve, O., … Andersson, S. (2018). Effect of higher vs standard dosage of vitamin D3 supplementation on bone strength and infection in healthy infants. *JAMA Pediatrics*, *172*(7), 646.

Roza, S. J., Van Lier, P. A. C., Jaddoe, V. W. V., Steegers, E. A. P., Moll, H. A., Mackenbach, J. P., … Tiemeier, H. (2008). Intrauterine growth and infant temperamental difficulties: The Generation R Study. *Journal of the American Academy of Child and Adolescent Psychiatry*, *47*(3), 264–272. https://doi.org/10.1097/CHI.0b013e318160b3df

Spry, E. A., Aarsman, S. R., Youssef, G. J., Patton, G. C., Macdonald, J. A., Sanson, A., … Olsson, C. A. (2020). Maternal and paternal depression and anxiety and offspring infant negative affectivity: A systematic review and meta-analysis. *Developmental Review*, *58*, 100934. https://doi.org/10.1016/j.dr.2020.100934

Stein, A., Pearson, R. M., Goodman, S. H., Rapa, E., Rahman, A., McCallum, M., … Pariante, C. M. (2014). Effects of perinatal mental disorders on the fetus and child. *The Lancet*, *384*, 1800–1819.

Taheriniya, S., Arab, A., Hadi, A., Fael, A., & Askari, G. (2021). Vitamin D and thyroid disorders: a systematic review and meta-analysis of observational studies. *BMC Endorcrine Disorders*, *21*, 171.

Vinkhuyzen, A. A. E., Eyles, D. W., Burne, T. H., Blanken, L. M. E., Kruithof, C. J., Verhulst, F., … McGrath, J. J. (2016). Prevalence and predictors of vitamin D deficiency based on maternal mid-gestation and neonatal cord bloods: The Generation R Study. *Journal of Steroid Biochemistry and Molecular Biology*, *164*, 161–167. https://doi.org/10.1016/j.jsbmb.2015.09.018

Vinkhuyzen, A. A. E., Eyles, D. W., Burne, T. H. J., Blanken, L. M. E., Kruithof, C. J., Verhulst, F., … McGrath, J. J. (2018). Gestational vitamin D deficiency and autism-related traits: the Generation R Study. *Molecular Psychiatry*, *23*(2), 240–246.
