# Supplementary material for: Prenatal maternal and cord blood vitamin D concentrations and negative affectivity in infancy
Source: Eur Child Adolesc Psychiatry. 2021 Oct 18;32(4):601–9. doi: 10.1007/s00787-021-01894-4 (PMC10115713; doi:10.1007/s00787-021-01894-4)
Supplement: Supplementary file 2 — Supplementary file2 (DOCX 42 KB) [file 787_2021_1894_MOESM2_ESM.docx]

**Prenatal maternal and cord blood vitamin D concentrations and negative affectivity in infancy**

**European Child & Adolescent Psychiatry**

Sara Sammallahti, Elisa Holmlund-Suila, Runyu Zou, Saara Valkama, Jenni Rosendahl, Maria Enlund-Cerullo, Helena Hauta-alus, Marius Lahti-Pulkkinen, Hanan El Marroun, Henning Tiemeier, Outi Mäkitie, Sture Andersson, Katri Räikkönen, Kati Heinonen

Corresponding author: Kati Heinonen, [kati.heinonen-tuomaala@tuni.fi](mailto:kati.heinonen-tuomaala@tuni.fi)

**Supplementary Tables**

[Supplementary Table 1. Associations between 25(OH)D concentrations measured during pregnancy and at birth and infant Negative Affectivity in VIDI and in the Generation R Study: basic models adjusting only for child sex and age at assessment. 2](#_Toc81230793)

[Supplementary Table 2. Comparison of analytical sample against cohort members who could not be included due to missing data (attrition group). 3](#_Toc81230794)

[Supplementary Table 3. Associations between 25(OH)D levels measured during pregnancy and at birth, and infant Negative Affectivity subscale scores in VIDI and in Generation R 5](#_Toc81230795)

## Supplementary Table 1. Associations between 25(OH)D concentrations measured during pregnancy and at birth and infant Negative Affectivity in VIDI and in the Generation R Study: basic models adjusting only for child sex and age at assessment.

|  |  | *EE (95% CI)* | *p* |
| --- | --- | --- | --- |
| **Maternal 25(OH)D in pregnancy** | |  |  |
|  | VIDI | -0.03 (-0.06, -0.01) | 0.02 |
|  | Generation R Study | -0.02 (-0.03, -0.003) | 0.01 |
| **Cord blood 25(OH)D at birth** | |  |  |
|  | VIDI | 0.00 (-0.02, 0.02) | 0.99 |
|  | Generation R Study | -0.02 (-0.04, -0.003) | 0.02 |

Abbreviations: 25(OH)D: 25-hydroxyvitamin D; EE: non-standardized effect estimate from linear regression model; CI: Confidence Interval for effect estimate; p: p-value

Effect estimates are presented as change in Negative Affectivity, in SD units, per each 10 nmol/L increase in 25(OH)D concentration. We adjusted for child sex and age at assessment.

| Supplementary Table 2. Comparison of analytical sample against cohort members who could not be included due to missing data (attrition group). | | | | | | | | | | | | | | | | | |
| --- | --- | --- | --- | --- | --- | --- | --- | --- | --- | --- | --- | --- | --- | --- | --- | --- | --- |
|  |  |  | **VIDI** | | | | | | |  | **Generation R** | | | | | | |
|  |  |  | **Analytical sample** | |  | **Attrition group** | |  | **Group difference** (p-value) |  | **Analytical sample** | |  | **Attrition group** | |  | **Group difference** (p-value) |
|  |  |  | *Total N=777* | |  | *Total N=198* | |  |  |  | *Total N=1,505* | |  | *Total N=2,254* | |  |  |
| ***Characteristics*** | | | Mean (SD) | N |  | Mean (SD) | N |  |  |  | Mean (SD) | N |  | Mean (SD) | N |  |  |
| **Maternal characteristics** | | |  |  |  |  |  |  |  |  |  |  |  |  |  |  |  |
|  | Pregnancy 25(OH)D, nmol/L | | 82.7 (20.4) | 651 |  | 78.2 (19.1) | 157 |  | 0.01 |  | 69.3 (28.2) | 1398 |  | 62.5 (30.1) | 1762 |  | <0.001 |
|  | Pregnancy 25(OH)D measurement season | |  | 651 |  |  | 157 |  | 0.85 |  |  | 1398 |  |  | 1762 |  | <0.001 |
|  |  | winter (Dec, Jan, Feb), n (%) | 144 (22.1%) |  |  | 38 (24.2%) |  |  |  |  | 290 (20.7) |  |  | 455 (25.8) |  |  |  |
|  |  | spring (Mar, Apr, May), n (%) | 97 (14.9%) |  |  | 21 (13.4%) |  |  |  |  | 372 (26.6) |  |  | 509 (28.9) |  |  |  |
|  |  | summer (Jun, Jul, Aug), n (%) | 168 (25.8%) |  |  | 37 (23.6%) |  |  |  |  | 403 (28.8) |  |  | 439 (24.9) |  |  |  |
|  |  | autumn (Sep, Oct, Nov), n (%) | 242 (37.2%) |  |  | 61 (38.9%) |  |  |  |  | 333 (23.8) |  |  | 359 (20.4) |  |  |  |
|  | Age at enrolment, years | | 31.2 (4.3) | 777 |  | 31.1 (5.0) | 102 |  | 0.81 |  | 31.6 (4.2) | 1505 |  | 30.2 (5.2) | 2254 |  | <0.001 |
|  | Early-pregnancy BMI, kg/m2 | |  | 777 |  |  | 114 |  | 0.24 |  |  | 1505 |  |  | 2247 |  | 0.30 |
|  |  | <18.5, n (%) | 21 (2.7%) |  |  | 7 (6.1%) |  |  |  |  | 29 (1.9) |  |  | 36 (1.6) |  |  |  |
|  |  | 18.5 to 24.9, n (%) | 575 (74.0%) |  |  | 80 (70.2%) |  |  |  |  | 1007 (66.9) |  |  | 1447 (64.4) |  |  |  |
|  |  | 25 to 29.9, n (%) | 135 (17.4%) |  |  | 19 (16.7%) |  |  |  |  | 339 (22.5) |  |  | 548 (24.4) |  |  |  |
|  |  | 30 or more, n (%) | 46 (5.9%) |  |  | 8 (7.0%) |  |  |  |  | 130 (8.6) |  |  | 216 (9.6) |  |  |  |
|  | Smoking, n (%) | | 109 (14.0%) | 777 |  | 21 (22.3%) | 94 |  | 0.045 |  | 330 (21.9) | 1505 |  | 635 (32.3) | 1966 |  | <0.001 |
|  | Educational level | |  | 777 |  |  | 105 |  | 0.06 |  |  | 1505 |  |  | 2244 |  | <0.001 |
|  |  | primary/secondary, n (%) | 189 (24.3%) |  |  | 37 (35.2%) |  |  |  |  | 407 (27.0) |  |  | 1012 (45.1) |  |  |  |
|  |  | lower tertiary, n (%) | 226 (29.1%) |  |  | 26 (24.8%) |  |  |  |  | 419 (27.8) |  |  | 512 (22.8) |  |  |  |
|  |  | upper tertiary, n (%) | 362 (46.6%) |  |  | 42 (40.0%) |  |  |  |  | 679 (45.1) |  |  | 720 (32.1) |  |  |  |
|  | BSI depression score >0.8, n (%) | | *NA* |  |  | *NA* |  |  |  |  | 53 (3.9) | 1374 |  | 130 (7.1) | 1836 |  | <0.001 |

*[continues on the next page]*

*[continued from the previous page]*

|  |  |  | **VIDI** | | | | | | |  | **Generation R** | | | | | | |
| --- | --- | --- | --- | --- | --- | --- | --- | --- | --- | --- | --- | --- | --- | --- | --- | --- | --- |
|  |  |  | **Analytical sample** | |  | **Attrition group** | |  | **Group difference** (p-value) |  | **Analytical sample** | |  | **Attrition group** | |  | **Group difference** (p-value) |
|  |  |  | *Total N=777* | |  | *Total N=198* | |  |  |  | *Total N=1,505* | |  | *Total N=2,254* | |  |  |
| ***Characteristics*** | | | Mean (SD) | N |  | Mean (SD) | N |  |  |  | Mean (SD) | N |  | Mean (SD) | N |  |  |
| **Infant characteristics** | | |  |  |  |  |  |  |  |  |  |  |  |  |  |  |  |
|  | Cord blood 25(OH)D, nmol/L | | 82.8 (26.3) | 763 |  | 76.5 (24.0) | 192 |  | 0.003 |  | 40.6 (20.5) | 1053 |  | 40.8 (22.2) | 1386 |  | 0.88 |
|  | Cord blood 25(OH)D measurement season | |  | 763 |  |  | 192 |  | 0.71 |  |  | 1053 |  |  | 1386 |  | <0.001 |
|  |  | winter (Dec, Jan, Feb), n (%) | 144 (18.9%) |  |  | 39 (20.3%) |  |  |  |  | 298 (28.3) |  |  | 280 (20.2) |  |  |  |
|  |  | spring (Mar, Apr, May), n (%) | 322 (42.2%) |  |  | 72 (37.5%) |  |  |  |  | 267 (25.4) |  |  | 317 (22.9) |  |  |  |
|  |  | summer (Jun, Jul, Aug), n (%) | 168 (22.0%) |  |  | 46 (24.0%) |  |  |  |  | 275 (26.1) |  |  | 417 (30.1) |  |  |  |
|  |  | autumn (Sep, Oct, Nov), n (%) | 129 (16.9%) |  |  | 35 (18.2%) |  |  |  |  | 213 (20.2) |  |  | 372 (26.8) |  |  |  |
|  | Sex, female, n (%) | | 398 (51.2%) | 777 |  | 90 (45.5%) | 198 |  | 0.15 |  | 751 (49.9) | 1505 |  | 1120 (49.7) | 2254 |  | 0.90 |
|  | Age at IBQ-R assessment, months, median [range] | | 11.7 [8.9, 16,1] | 777 |  | 11.9 [11.0, 14.0] | 39 |  | 0.01 |  | 6.2 [4.7, 11.9] | 1505 |  | 6.4 [5.6, 11.4] | 254 |  | 0.02 |
|  | IBQ-R Negative affectivity score, SD units | | 0.00 (0.7) | 777 |  | -0.03 (0.8) | 49 |  | 0.80 |  | -0.2 (0.7) | 1505 |  | -0.3 (0.7) | 254 |  | 0.06 |

Abbreviations: BDI: Beck Depression inventory; BMI: body-mass-index; IBQ-R: Infant Behavior Questionnaire, Revised; N: number of participants with available data; n: number of cases; NA: Data not available in the cohort in question; p-value: p-value from non-response analyses in which the analytical sample was compared against those cohort members who could not be included in the study because of missing data, using independent t-tests (normally distributed variables), Mann-Whitney U tests (non-normally distributed continuous variables), and chi-squared tests (categorical variables); SD: standard deviation; %: proportion of cases among those with data available

##

## Supplementary Table 3. Associations between 25(OH)D levels measured during pregnancy and at birth, and infant Negative Affectivity subscale scores in VIDI and in Generation R

| **Panel A. Sadness subscale** | |  |  |  |  |  |
| --- | --- | --- | --- | --- | --- | --- |
|  |  | ***Model I*** | |  | ***Model II*** | |
|  |  | EE (95% CI) | *p-value* |  | EE (95% CI) | *p-value* |
| *Maternal 25(OH)D in pregnancy* | |  |  |  |  |  |
|  | Within VIDI | -0.04 (-0.08, 0.00) | 0.06 |  | -0.04 (-0.08, 0.00) | 0.06 |
|  | Within Generation R | -0.02 (-0.03, 0.003) | 0.10 |  | -0.02 (-0.04, -0.0004) | 0.05 |
| *Cord blood 25(OH)D at birth* | |  |  |  |  |  |
|  | Within VIDI | 0.01 (-0.02, 0.04) | 0.55 |  | 0.01 (-0.02, 0.04) | 0.51 |
|  | Within Generation R | -0.01 (-0.04, 0.02) | 0.42 |  | -0.02 (-0.05, 0.01) | 0.18 |
| **Panel B. Distress to limitations subscale** | | |  |  |  |  |
|  |  | ***Model I*** | |  | ***Model II*** | |
|  |  | EE (95% CI) | *p-value* |  | EE (95% CI) | *p-value* |
| *Maternal 25(OH)D in pregnancy* | |  |  |  |  |  |
|  | Within VIDI | -0.04 (-0.08, 0.00) | 0.048 |  | 0.04 (-0.08, 0.00) | 0.05 |
|  | Within Generation R | -0.02 (-0.04, -0.002) | 0.03 |  | -0.02 (-0.04, -0.002) | 0.03 |
| *Cord blood 25(OH)D at birth* | |  |  |  |  |  |
|  | Within VIDI | 0.01 (-0.02, 0.04) | 0.45 |  | 0.01 (-0.02, 0.04) | 0.47 |
|  | Within Generation R | -0.03 (-0.05, 0.004) | 0.09 |  | -0.03 (-0.06, -0.001) | 0.046 |
| **Panel C. Fearfulness subscale** | |  |  |  |  |  |
|  |  | ***Model I*** | |  | ***Model II*** | |
|  |  | EE (95% CI) | *p-value* |  | EE (95% CI) | *p-value* |
| *Maternal 25(OH)D in pregnancy* | |  |  |  |  |  |
|  | Within VIDI | -0.02 (-0.05, 0.02) | 0.43 |  | -0.01 (-0.05, 0.03) | 0.58 |
|  | Within Generation R | -0.01 (-0.02, 0.01) | 0.37 |  | -0.01 (-0.03, 0.01) | 0.40 |
| *Cord blood 25(OH)D at birth* | |  |  |  |  |  |
|  | Within VIDI | -0.02 (-0.06, 0.01) | 0.13 |  | 0.02 (-0.06, 0.01) | 0.16 |
|  | Within Generation R | -0.02 (-0.05, 0.01) | 0.14 |  | -0.02 (0.05, 0.005) | 0.10 |
| **Panel D. Recovery from distress subscale (inverse-coded)** | | | |  |  |  |
|  |  | ***Model I*** | |  | ***Model II*** | |
|  |  | EE (95% CI) | *p-value* |  | EE (95% CI) | *p-value* |
| *Maternal 25(OH)D in pregnancy* | |  |  |  |  |  |
|  | Within VIDI | -0.05 (-0.08, -0.01) | 0.02 |  | 0.05 (-0.09, -0.01) | 0.02 |
|  | Within Generation R | -0.02 (-0.04, -0.004) | 0.02 |  | -0.03 (-0.05, -0.01) | 0.01 |
| *Cord blood 25(OH)D at birth* | |  |  |  |  |  |
|  | Within VIDI | 0.01 (-0.02, 0.04) | 0.58 |  | 0.00 (-0.03, 0.03) | 0.93 |
|  | Within Generation R | -0.03 (-0.06, -0.01) | 0.02 |  | -0.04 (-0.08, -0.01) | 0.01 |

Model I: adjusted for child sex and age at assessment. Model II: adjusted for Model I covariates and further for maternal age, educational level, early-pregnancy BMI, and smoking, and season of vitamin D measurement

Abbreviations: EE: non-standardised effect estimate from linear regression model; CI: Confidence Interval for effect estimate
